# Impact of Emergency Warning Nursing on CRP, PCT, TNF-α and Clinical Indicators in Patients with Acute Stress Disorder under Hierarchical Analysis

**DOI:** 10.62641/aep.v53i1.1685

**Published:** 2025-01-05

**Authors:** Yanxia Shao, Xiaoping Zhou, Han Liu, Tianjiao Li, Yushu Wang, Ya Chen, Xiangcheng Huang, Wei Sun

**Affiliations:** ^1^Emergency Department, The Southwest Hospital of AMU, 400038 Chongqing, China; ^2^Rehabilitation Department, The Southwest Hospital of AMU, 400038 Chongqing, China; ^3^Clinical Teaching and Research Department, The Southwest Hospital of AMU, 400038 Chongqing, China

**Keywords:** Acute Stress Disorder, Analytic Hierarchy Process, emergency warning, inflammatory factors, psychological resilience, posttraumatic growth

## Abstract

**Background:**

In emergency warning nursing, the pre-alert system significantly influences the biochemical markers and clinical outcomes of patients with Acute Stress Disorder. Therefore, this study applies hierarchical analysis to explore the impact of early warning nursing on crucial indicators such as C-reactive protein (CRP), procalcitonin (PCT), tumor necrosis factor-alpha (TNF-α), interleukin-6 (IL-6), and assess their clinical efficacy.

**Methods:**

The study selected patients with acute stress disorder who were hospitalized in Southwest Hospital of Chongqing from December 2021 to December 2022, and collected data from 250 patients. Through PSM score matching, 170 patients were finally scored and grouped, 85 patients in each group, which were divided into routine group and stratified analysis group. The changes in serum inflammatory markers, psychological resilience, and post-traumatic growth were compared between the two experimental groups on day 1 of admission and after 14 days of intervention.

**Result:**

After one day of admission, there was no significant difference in the serum factor levels, psychological resilience, and post-traumatic growth among the participants (*p* > 0.05). However, after 14 days of intervention, patients in the hierarchical analysis group showed better outcomes in serum inflammatory markers such as C-reactive protein, procalcitonin, tumor necrosis factor-alpha, and interleukin-6 compared to the conventional group (*p* < 0.05). The hierarchical analysis group had higher psychological resilience scores regarding strength, optimism, and resilience compared to the conventional group (*p* < 0.05). Furthermore, the hierarchical analysis group showed higher post-traumatic growth scores regarding mental changes, personal strength, appreciation of life, interpersonal relationship, and new possibilities relative to the conventional group (*p* < 0.05).

**Conclusion:**

Analytic Hierarchy Process (AHP)-based emergency warning nursing can help improve the serum inflammatory factor levels, strengthen psychological resilience, and enhance post-traumatic growth levels in patients with Acute Stress Disorder.

## Introduction

Acute Stress Disorder, a psychological condition induced by intense or persistent stressors, often manifests with symptoms such as anxiety, fear, and depression, and may be linked to abnormal physiological indicators. Common inflammatory and immune-related markers, including C-reactive protein (CRP), procalcitonin (PCT), tumor necrosis factor-alpha (TNF-α), and interleukin-6 (IL-6), may significantly affect the onset and progression of Acute Stress Disorder. Emergency early warning is a specialized nursing approach designed for patients with Acute Stress Disorder, aiming to mitigate stress responses and promote recovery through timely alerts and interventions. Furthermore, the Analytic Hierarchy Process is a decision-making method that breaks down problems into multiple levels and factors, comparing their relative importance to provide a scientific basis for decision-making.

Acute stress events can induce the activation of the immune system, resulting in the release of inflammatory responses and an increase in the production of inflammatory factors such as C-reactive protein, procalcitonin, tumor necrosis factor-alpha, and interleukin-6 [1]. Acute Stress Disorder is a mental health condition that occurs after a traumatic event, and is characterized by clinical symptoms like increased alertness, persistent emotional numbness, insomnia, and various cognitive, emotional, and behavioral issues. In severe cases, individuals may experience social withdrawal, cognitive disorders, and even engage in self-harm [2]. Acute Stress Disorder is a common mental illness worldwide, with a post-traumatic probability of around 5% to 20%, while in China, the incidence rate ranges from 10% to 30% [3]. Recently, Acute Stress Disorder research has received international attention. Functional magnetic resonance imaging (fMRI) has demonstrated that the brain activity patterns in patients with Acute Stress Disorder differ from those with post-traumatic stress disorder (PTSD) [4]. Cognitive behavioral therapy (CBT) and eye movement desensitization and reprocessing (EMDR) have been found to be effective therapies for sufferers with Acute Stress Disorder [5]. Furthermore, abnormal levels of inflammatory factors and neurotransmitters are suggested to be associated with the occurrence and severity of Acute Stress Disorder [6].

C-reactive protein (CRP) is an inflammatory marker produced during infection or tissue damage in the body. As an acute-phase protein, its concentration can increase significantly in a short period [7]. Procalcitonin (PCT), a precursor protein of endogenous calcitonin, is typically synthesized by C cells and other cells. Tumor necrosis factor-alpha (TNF-α) is a crucial inflammatory mediator that substantially influences inflammation and immune responses. Interleukin-6 (IL-6) is another vital inflammatory mediator and cellular signaling molecule that plays a pivotal role in immune and inflammatory responses [8]. Therefore, understanding the impact of emergency department nursing on these inflammatory factors and other clinical indicators is essential for optimizing the treatment and rehabilitation of patients with Acute Stress Disorder.

Analytic Hierarchy Process is a systematic research method that achieves significant results by analyzing and evaluating the relative importance of multiple factors through comprehensive weight analysis [9]. Comprehensively assessing various levels of emergency care interventions can provide a thorough understanding of their impact on inflammatory factor levels and clinical indicators. The biomarkers such as C-reactive protein, procalcitonin, tumor necrosis factor-alpha, and interleukin-6 have been assessed in Acute Stress Disorder, indicating correlations with stress responses and inflammatory activity. However, it is important to note that although this study has provided valuable insights into the physiological and pathological processes of stress, the complexity and individual variability of Acute Stress Disorder necessitate more comprehensive research for a deeper understanding.

Therefore, this study aims to assess the effectiveness of emergency early warning in patients with Acute Stress Disorder and to conduct a detailed analysis of the changes in specific biochemical markers and their impact on clinical indicators through hierarchical analysis. Particularly, it explores the effects of emergency early warning nursing on C-reactive protein, procalcitonin, tumor necrosis factor-alpha, and other clinical indicators in patients with Acute Stress Disorder using Analytic Hierarchy Process.

## Materials and Methods

### Selection of the Study Subjects

Basic data of hospitalized patients with acute stress disorder in the First Affiliated Hospital of China Army Military Medical University (Chongqing Southwest Hospital) from December 2021 to December 2022 were collected as the observation objects. The basic data of 250 patients were preliminarily collected. After matching with PSM score, 170 patients were statistically scored and grouped according to the ratio of 1:1. They were divided into routine group and stratified analysis group with 85 cases in each group. Informed consent forms were signed by either the patients or their family members. Inclusion criteria for the patients were set as follows: ① patients who meet the diagnostic criteria for Acute Stress Disorder based on the Chinese Classification and Diagnosis of Mental Disorders-3 (CCMD-3) [10], ② with first-time onset, and ③ individuals over 18 years old. Furthermore, exclusion criteria were as below: ① patients with active bleeding in the brain, chest, and other areas, ② those with dysfunction or incomplete function of the brain, lungs, heart, and other organs, ③ individuals with a previous history of taking antipsychotic drugs, and ④ those with dementia, schizophrenia, and other conditions who are unable to communicate independently with healthcare professionals. This study conforms to the Declaration of Helsinki and is approved by the Ethics Committee of the First Affiliated Hospital of China Army Military Medical University (Chongqing Southwest Hospital)(Approval No.: (A) KY2023045).

### Assessment of the Study Subjects

#### Conventional Group 

The nursing staff were directed to assess the patients according to the given study design. Routine psychological nursing measures must be implemented in the emergency department. Nursing staff must conduct a comprehensive physical and mental assessment of patients, including understanding the nature and severity of the event, the patient’s symptoms and reactions, current mental health status, and observing the patient’s emotional state, anxiety level, sleep quality, and behavioral performance. Furthermore, patients should be provided with a safe, quiet, and comfortable environment to alleviate their stimulation and stress, ensure their privacy and respect, and avoid further triggering factors. The nursing staff should establish good communication and trust with patients, listening to their needs and feelings, which are crucial components in patient care. The patients and their families should be educated and informed about Acute Stress Disorder, including symptoms, self-management skills, and the rehabilitation process. It is essential to explain the common and short-term nature of symptoms and provide positive prospects and hope. Additionally, the patients should be offered a comfortable position and, if necessary, appropriate sedative or soothing medication, such as pain relievers, as instructed by a doctor.

#### Hierarchical Analysis Group

Emergency warning and nursing measures were implemented in this group of patients using the Analytic Hierarchy Process. In addition to conventional emergency warning nursing measures, Analytic Hierarchy Process-based emergency warning nursing measures were integrated into this group.

Risk Evaluation Criteria of Analytic Hierarchy Process

With the assistance of authoritative experts in the department of psychology, responsible nurses use the post-traumatic stress disorder checklist version (PCL-C), which mainly includes three dimensions: increased alertness (5 items), numbness/avoidance (7 items), and re-experience (5 items). There are a total of 17 items, with each item scoring up to 5 points, resulting in a total score of 0–85 points. Mild psychological disorder is represented by 17–37 points, Moderate psychological disorder by 38–49 points, and severe psychological disorder by >50 points. The Cronbach’s α coefficient for the scale is 0.941. A higher score indicates a greater degree of psychological trauma stress [11]. Therefore, it is necessary to develop different modules of psychological stress reserve improvement plans based on the characteristics and personal needs of various types of trauma patients.

Emergency Department Early Warning Nursing Content

① Mild: The nursing staff assist the patients in relaxation through a deep breathing process. Deep breathing techniques include guiding the patients to practice deep breathing exercises in a quiet and comfortable environment to assist in relaxing their tense bodies and reducing anxiety. Relevant personnel assist the patient in finding a comfortable flat or semi-seated position, ensuring that their body and limbs are completely relaxed. Furthermore, they are advised to inhale slowly through the nose, expanding the chest while paying attention to the sensation of air entering the nasal cavity and lungs. The patients are instructed to exhale slowly through the mouth, shrinking the chest, and are directed to count to 3 or 4 silently between each inhalation and exhalation. The patients are advised to repeat this cycle 20 to 30 times each session, once daily. Additionally, patients are encouraged to engage in multiple deep breathing exercises, gradually increasing the number and duration of exercises based on their needs and abilities.

Furthermore, nursing staff help the patients with progressive muscle relaxation. They assist patients in a quiet and comfortable environment, instructing them into a relaxed position. The sequences of muscle relaxation are as follows: feet, calves, thighs, buttocks, abdomen, chest, back, upper limbs, hands, neck, and face. For each body part, the patients are instructed to tighten the muscles for three seconds, then relax them for three seconds. Additionally, the patients are directed to focus on each body part, relaxing each position 10–15 times. Each training session lasts 15–20 minutes and is practiced twice daily.

② Moderate: Based on deep breathing and gradual psychological relaxation approaches, nursing staff, under the guidance of a psychiatrist, choose soft, gentle, and soothing music, such as natural sounds, light music, and meditation music, from a music library according to the patient’s personal preferences. The volume should be controlled at 20% to 30% to ensure that the music does not overly stimulate the patient or cause discomfort. Furthermore, the nursing staff guide patients into a meditative state using a calm and gentle voice, encouraging them to listen to the sound and melody of music to experience the emotions and sensations. Moreover, patients are directed to focus on their breathing, bodily sensations, and the melody of the music to maintain a calm and relaxed state of mind.

③ Severe: The nursing staff assist the patients using a cognitive reconstructing approach to change their way of thinking. They provide detailed explanations about Acute Stress Disorder to patients and their families, including symptoms, onset process, possible causes, and other necessary information to help them better understand the condition. They inform them that their symptoms are common psychological reactions. Nursing staff encourage sufferers to express their experiences, including feelings and reactions to traumatic events, within a safe and supportive environment where they can feel understood and accepted. Furthermore, they assist patients in identifying and challenging negative perceptions related to traumatic events, such as over-blaming, over-interpreting threats, and being pessimistic about the future. They help patients in analyzing and evaluating evidence of negative cognition, guiding them to consider whether there is sufficient evidence to support their negative thinking or if there may be other more reasonable explanations. Additionally, they encourage patients to find alternative explanations or ways to interpret traumatic events.

Furthermore, staff assist patients in reducing their anxiety and fear using a systematic desensitization method. They actively communicate with patients and their families to understand and grasp the stressors causing anxiety or fear. They identify the stimuli or situations that require desensitization treatment and develop a gradually increasing exposure plan based on the patient’s ability and comfort level. Patients start with lower levels of direct stimulation and gradually increase to more challenging stimuli to help them adapt and overcome fear and anxiety. Through gradual exposure, they guide patients to face fear and anxiety. For example, they might start by searching for images of scenes similar to their stressors, then gradually move to video playback and scenario simulation as they become comfortable. Family members are instructed to support the patients through systematic desensitization therapy sessions, lasting 20–30 minutes each, 2–3 times a week.

### Observation Items and Evaluation Criteria

#### Serum Inflammatory Factors (SIF)

After one day of admission and 14 days of intervention, the nursing staff observed and analyzed the levels of inflammatory factor indicators, such as C-reactive protein normal range (NR): 0.8–8 mg/L (Catalog No. IPD10062H), procalcitonin <0.05 ng/mL (Catalog No. IPD10065H), tumor necrosis factor-alpha normal range: 0.74–1.54 ng/mL (Catalog No. F110TA04), interleukin-6 normal range: 0.373–0.463 ng/L (Catalog No. HY-P700749), using intravenous enzyme-linked immunosorbent assay (ELISA) (Shanghai Jianglai Biotechnology Co., Ltd., part number JL15135).

#### Psychological Resilience

After one day of admission and 14 days of intervention, the nursing staff used the Chinese version of the Connor Davidson Resilience Scale (CD-RISC), developed by Connor in 2003 [12]. This scale mainly includes three dimensions: strength (0–72 points), optimism (0–16 points), and resilience (0–12 points). There are a total of 25 items, with a total score of 0–100 points. A higher score indicates greater psychological resilience. The Cronbach’s α coefficient for CD-RISC is 0.91.

#### Assessing Post-Traumatic Growth

After one day of admission and 14 days of intervention, nursing staff used the Post-Traumatic Growth Inventory (PTGI) developed by Wang ji in 2011 [13]. This scale includes 5 dimensions: mental change 5, personal strength (PS) 4, appreciation of life (AOL) 4, interpersonal relationships (IR) 4, and new possibilities (NP) 4. There are a total of 21 items, with a maximum score of 5 points for each item and a total score of 0–105 points. Higher scores indicate more significant post-traumatic growth.

### Data Analysis

Statistical analysis was performed using SPSS 26.0 software (Manufacturer: IBM Corporation, City: Armonk, NY, Country: USA). The measurement data with normal distribution were expressed as mean ± standard deviation ($\bar{x}$ ± s), and a *t*-test was used for inter-group comparison (IGC). Paired *t*-tests were used for intra-group comparisons. The count data were expressed as frequencies and analyzed by the chi-square test. A *p* < 0.05 was considered statistically significant.

## Result

### Comparison of Baseline Data

The basic data of 250 patients were collected, there was no significant difference between the two groups before matching (*p* > 0.05). After matching the PSM score, 170 patients were statistically scored and grouped according to the ratio of 1:1. They were divided into routine group and stratified analysis group, 85 cases in each group. The comparison of baseline data, such as gender, age, educational level, type of trauma, body mass index, and Acute Physiology and Chronic Health Evaluation II (APACHE II) between the two groups showed no significant differences (*p* > 0.05, Table 1).

**Table 1.** **Comparison of clinical basic data between the two groups before and after propensity matching**.

Experimental groups	Gender (male/female)	Age ($\bar{x}$ ± s, year)	Education level (high school and below/college and above)	Type of trauma (traffic accident/fall from height/others)	Body mass index ($\bar{x}$ ± s, kg/m^2^)	APACHE II ($\bar{x}$ ± s, scores)
Hierarchical analysis group (*n* = 130)	79/51	59.61 ± 4.34	76/54	48/51/31	21.32 ± 1.44	25.53 ± 1.44
Conventional group (*n* = 120)	65/55	59.63 ± 4.29	64/56	45/48/27	21.61 ± 1.17	25.68 ± 1.28
χ ^2^ */t*	1.114	0.037	0.666	0.064	1.739	0.868
*p*	0.291	0.971	0.414	0.969	0.083	0.386
Hierarchical analysis group (*n* = 85)	48/37	58.53 ± 3.44	46/39	28/31/26	21.50 ± 1.29	25.49 ± 1.33
Conventional group (*n* = 85)	46/39	58.46 ± 3.53	44/41	26/33/26	21.55 ± 1.18	25.61 ± 1.38
χ ^2^ */t*	0.095	0.131	0.094	0.137	0.264	0.577
*p*	0.758	0.896	0.759	0.934	0.792	0.565

APACHE II, Acute Physiology and Chronic Health Evaluation II.

### Comparison of Serum Inflammatory Factors

**Fig. 1.**
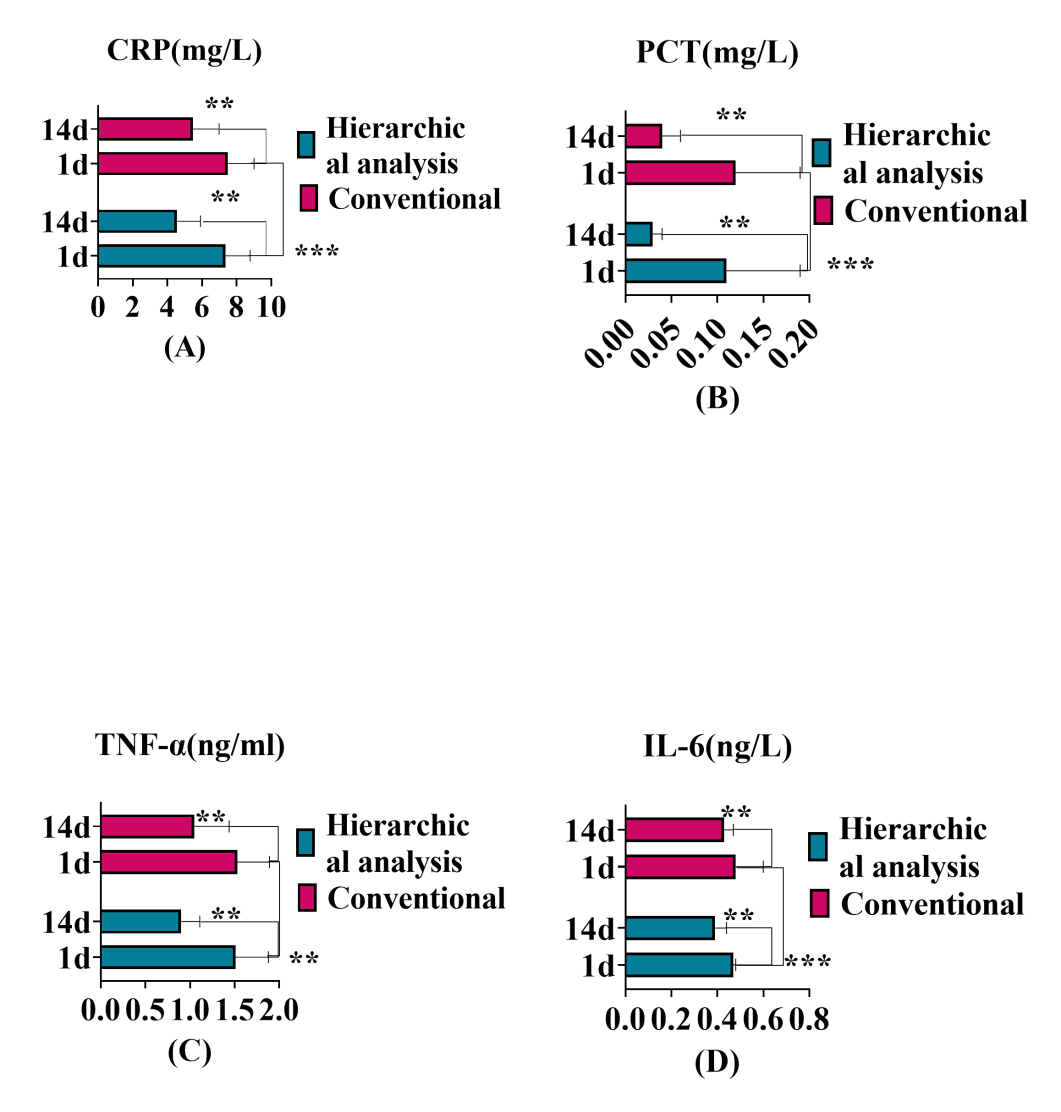
**Comparison of SIF levels between the two groups before and after intervention**. Notes: (A) C-reactive protein; (B) Procalcitonin; (C) Tumor necrosis factor-alpha; (D) Interleukin-6. “^**^” indicates *p* < 0.01; “^*⁣**^” indicates *p* < 0.001; hierarchical analysis group (n = 85); conventional group (n = 85); X-axis: the serum inflammatory factor index level; Y axis: the intervention time, which is one day and 14 days after admission. SIF, serum inflammatory factors; CRP, C-reactive protein; PCT, procalcitonin; TNF-α, tumor necrosis factor-alpha; IL-6, interleukin-6.

After one day of admission, there was no significant difference in serum factor levels between the two groups (*p* > 0.05). However, after 14 days of intervention, patients in the hierarchical analysis group showed lower serum inflammatory markers levels, such as C-reactive protein, procalcitonin, tumor necrosis factor-alpha, and interleukin-6 compared to the conventional group (*p* < 0.05). The levels of serum inflammatory factors are shown in Table 2 and Fig. 1.

**Table 2.** **Comparison of serum inflammatory factors levels before and after intervention**.

Experimental groups	CRP (mg/L)		PCT (mg/L)		TNF-α (ng/mL)		IL-6 (ng/L)	
	Admission for 1 day	After 14 days of intervention	Admission for 1 day	After 14 days of intervention	Admission for 1 day	After 14 days of intervention	Admission for 1 day	After 14 days of intervention
Hierarchical analysis group (*n* = 85)	7.36 ± 1.42	4.55 ± 1.36^**^	0.11 ± 0.08	0.03 ± 0.01^**^	1.51 ± 0.37	0.90 ± 0.21^**^	0.47 ± 0.01	0.39 ± 0.05^**^
Conventional group (*n* = 85)	7.48 ± 1.53	5.47 ± 1.52^**^	0.12 ± 0.07	0.04 ± 0.02^**^	1.53 ± 0.36	1.05 ± 0.39^**^	0.48 ± 0.12	0.43 ± 0.04^**^
*t*-value	0.530	4.159	0.867	4.123	0.357	3.122	0.766	5.759
*p*-value	0.597	<0.001	0.387	<0.001	0.721	0.002	0.445	<0.001

Note: Compared with the same group admitted for 1 day, ^**^p < 0.01. CRP, C-reactive protein; PCT, procalcitonin; TNF-α, tumor necrosis factor-alpha; IL-6, interleukin-6.

### Psychological Resilience

**Fig. 2.**
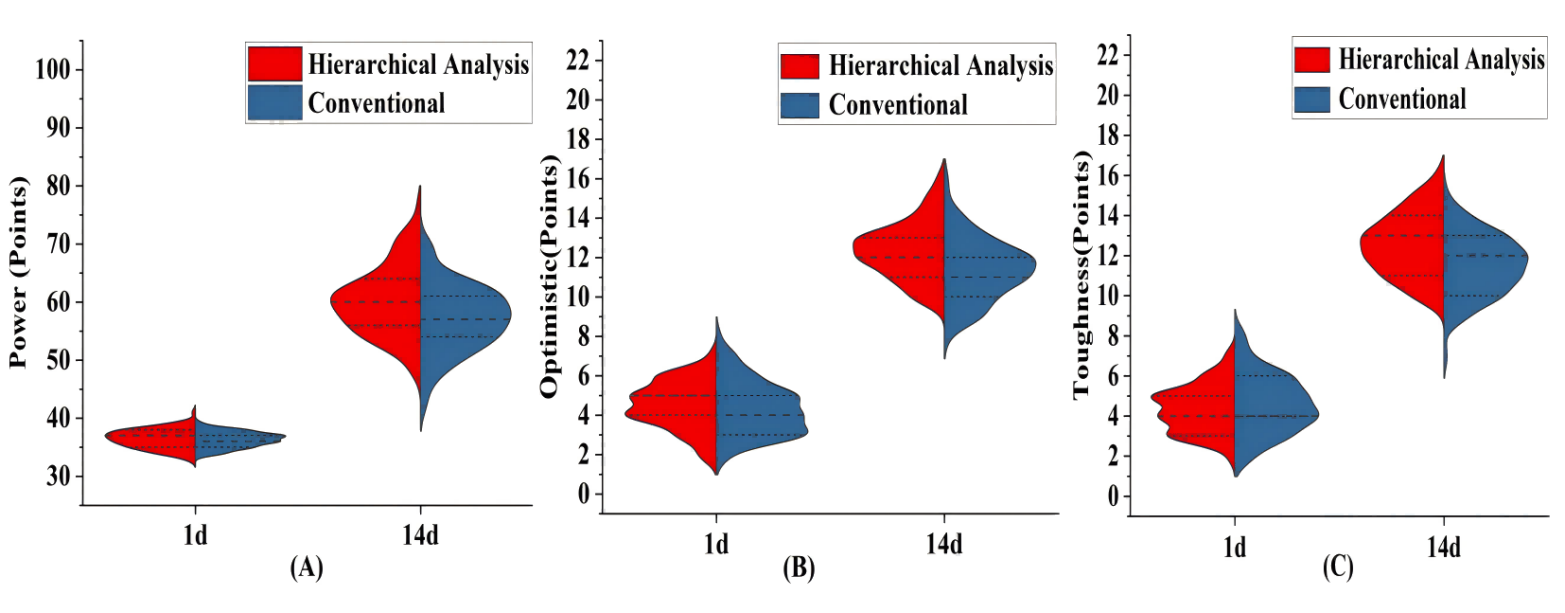
**Comparison of scores for various indicators of psychological resilience among the participants before and after intervention**. Notes: (A) shows the force. (B) indicates optimistic. (C) shows toughness. Hierarchical analysis group (n = 85); conventional group (n = 85); X-axis: represents the intervention time, which is 1 day and 14 days after admission; Y axis: indicates the score of each resilience dimension; Violin Shape: shows the distribution of scores for each group, with width representing density and height representing quantity; Black wire inside: is the median of the kernel density estimate; External black wire: is the range of quartiles; Time comparison: from the left to right of the figure, the progression in treatment time can be seen.

After 1 day of admission, there were no significant changes in the psychological resilience scores of the participants (*p* > 0.05). However, after 14 days of intervention, the hierarchical analysis group showed higher psychological resilience regarding of strength, optimism, and resilience compared to the conventional group (*p* = 0.001, Table 3 and Fig. 2).

**Table 3.** **Comparison of participants before and after CD-RISC intervention [($\bar{x}$ ± s), points]**.

Experimental groups	Power		Optimistic		Toughness	
	After one day of admission	After 14 days of intervention	After one day of admission	After 14 days of intervention	After one day of admission	After 14 days of intervention
Hierarchical analysis group (*n* = 85)	36.61 ± 1.46	59.31 ± 5.43^**^	4.45 ± 1.36	12.29 ± 1.53^**^	4.53 ± 1.35	12.37 ± 1.65^**^
Conventional group (*n* = 85)	36.38 ± 1.33	56.56 ± 5.39^**^	4.52 ± 1.47	11.48 ± 1.66^**^	4.68 ± 1.47	11.58 ± 1.40^**^
*t*-value	1.074	3.314	0.322	3.308	0.693	3.366
*p*-value	0.285	0.001	0.748	0.001	0.489	0.001

Note: Compared with the same group admitted for 1 day, ^**^p < 0.01. CD-RISC, Connor Davidson Resilience Scale.

### Post-Traumatic Growth

**Fig. 3.**
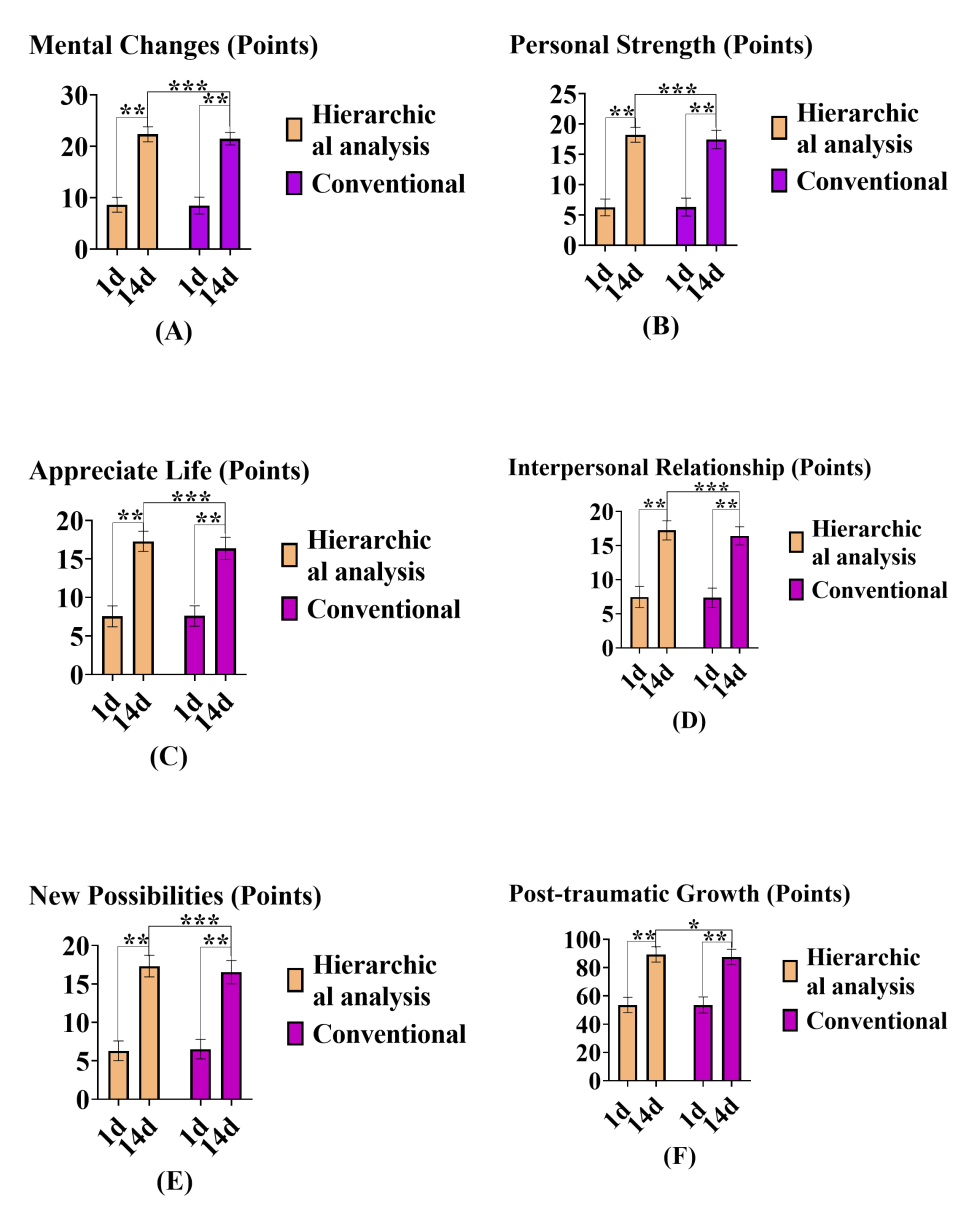
**Comparison of post-traumatic growth scores between the two groups before and after intervention**. Notes: (A) Mental changes. (B) Individual power. (C) Appreciate life. (D) Interpersonal relationships. (E) New possibilities. (F) Post-traumatic growth. “^*^” means *p* < 0.05; “^**^” means *p* < 0.01; “^*⁣**^” means *p* < 0.001. Hierarchical analysis group (n = 85); conventional group (n = 85); X-axis: represents the intervention time, which is 1 day and 14 days after admission; Y axis: shows the post-traumatic growth dimension score and total score.

After one day of admission, there was no significant difference in the post-traumatic growth scores among the participants (*p* > 0.05). However, after 14 days of intervention, the hierarchical analysis group showed higher post-traumatic growth scores in terms of mental changes, PS, AOL, IR, and NP compared to the convention group (*p* < 0.05). A comparison of post-traumatic growth scores is shown in Table 4 and Fig. 3.

**Table 4.** **Comparison of trauma growth among the participants before and after intervention [($\bar{x}$ ± s), points]**.

Experimental groups	Mental changes		Personal power		Appreciate life	
	After one day of admission	After 14 days of intervention	After one day of admission	After 14 days of intervention	After one day of admission	After 14 days of intervention
Hierarchical analysis group (*n* = 85)	8.63 ± 1.44	22.35 ± 1.46^**^	6.25 ± 1.38	18.23 ± 1.21^**^	7.55 ± 1.36	17.29 ± 1.31^**^
Conventional group (*n* = 85)	8.47 ± 1.63	21.49 ± 1.23^**^	6.32 ± 1.47	17.44 ± 1.52^**^	7.60 ± 1.33	16.37 ± 1.44^**^
*t*-value	0.678	4.153	0.320	3.749	0.242	4.357
*p*-value	0.499	<0.001	0.749	<0.001	0.809	<0.001
Experimental groups	Interpersonal relationship		New possibilities		Post-traumatic growth	
	Admission for 1 day	After 14 days of intervention	Admission for 1 day	After 14 days of intervention	Admission for 1 day	After 14 days of intervention
Hierarchical analysis group (*n* = 85)	7.47 ± 1.55	17.25 ± 1.41^**^	6.31 ± 1.28	17.33 ± 1.41^**^	53.57 ± 5.47	89.37 ± 5.41^**^
Conventional group (*n* = 85)	7.35 ± 1.43	16.44 ± 1.33^**^	6.52 ± 1.27	16.54 ± 1.52^**^	53.63 ± 5.66	87.53 ± 5.49^**^
*t*-value	0.523	3.853	1.074	3.513	0.070	2.201
*p*-value	0.601	<0.001	0.285	<0.001	0.944	0.029

Note: Compared with the same group admitted for 1 day, ^**^p < 0.01.

## Discussion

This study observed that emergency warning nursing using the Analytic Hierarchy Process diminishes the levels of inflammatory factors in sufferers with acute emergency disorders (*p* < 0.05). Analytic Hierarchy Process-based emergency early warning nursing helps alleviate patients’ psychological pressure and emotional distress, positively impacting their inflammatory factor levels [14, 15]. Moreover, emotional stress and psychological distress can alter the neuroendocrine and immune systems, increasing the secretion of corticosteroids such as cortisol and leading to excessive activation and elevation of inflammatory factors [16]. By providing intervention measures such as emotional support, psychological counseling, and cognitive behavioral therapy, emergency early warning nursing can restore patients’ psychological balance, improve their psychological and emotional state, and enhance the activity and function of immune cells [17]. Furthermore, achieving emotional stability and psychological relaxation helps to balance the activity of the immune system and reduce the production and release of SIF such as C-reactive protein, procalcitonin, and tumor necrosis factor-alpha [18].

Our findings revealed that compared to patients receiving routine emergency department nursing intervention, those in the observation group receiving combined nursing intervention exhibited a significant increase in psychological resilience (*p* < 0.05). Acute Stress Disorder causes rapid emotional fluctuations in response to external and internal stress stimuli. To manage traumatic memories and triggers, patients may avoid people, places, or activities related to the event, attempting to reduce emotional burden by avoiding recalling traumatic experiences, thereby alleviating their psychological resilience level [19]. Nursing staff promote patients’ understanding and mastery of stress reactions by providing psychological education and cognitive reconstructing approaches along with coping strategies and skills. This technique increases patients’ awareness of their psychological resilience and cultivates their ability to respond to stress actively [20, 21]. Through relaxation training, patients can better respond to their emotional states, recover from them, and enhance their self-confidence and emotional regulation ability, thereby promoting psychological resilience [22, 23].

Additionally, this study found that the post-traumatic growth level of sufferers with Acute Stress Disorder in the conventional group significantly exceeded compared to that in the control group (*p* < 0.05). By discussing and guiding patients to think about their trauma, they can gradually find positive meaning and possibilities for growth, thereby improving their level of post-traumatic growth [24]. Nursing staff carefully listen to the emotional needs of patients and provide appropriate psychological support, encouraging patients to fully express their inner experiences and feelings. This process promotes emotional release and regulation, creating conditions for post-traumatic growth. Furthermore, they encourage and assist patients in establishing a positive self-image, rebuilding goals and values, and gradually restoring confidence and self-esteem, thereby promoting the development of post-traumatic growth.

Despite some promising findings, this study has certain limitations. There may be errors or deviations in the measurement methods of biochemical indicators such as C-reactive protein, procalcitonin, tumor necrosis factor-alpha, and interleukin-6, affecting the accuracy of the results. Inappropriate or simplistic statistical methods may not fully reveal the impact of emergency pre-warning care on these indicators and their complex relationship. Due to the limitations of the study design, the conclusions may only apply to specific populations or situations and might not be widely generalized to other patients or clinical settings. Furthermore, the explanation and discussion of the study results may not be in-depth enough to fully expound the mechanism and possible reasons for the impact of emergency early warning nursing on various indicators.

## Conclusion

In conclusion, Analytic Hierarchy Process-based emergency early warning nursing has a positive influence on improving SIF levels and strengthening the psychological resilience of patients with Acute Stress Disorder while also enhancing their post-traumatic growth level. This approach positively impacts the short-term and long-term physical recovery of the patients.

## Availability of Data and Materials

The datasets used and/or analyzed during the current study are available from the corresponding author upon reasonable request.

## References

[1] Shahrour G, Dardas LA (2020). Acute Stress Disorder, coping self-efficacy and subsequent psychological distress among nurses amid COVID-19. *Journal of Nursing Management*.

[2] Thakur A, Choudhary D, Kumar B, Chaudhary A (2022). A Review on Post-traumatic Stress Disorder (PTSD): Symptoms, Therapies and Recent Case Studies. *Current Molecular Pharmacology*.

[3] Ye Z, Yang X, Zeng C, Wang Y, Shen Z, Li X (2020). Resilience, Social Support, and Coping as Mediators between COVID-19-related Stressful Experiences and Acute Stress Disorder among College Students in China. *Applied Psychology. Health and Well-being*.

[4] Tarsitani L, Vassalini P, Koukopoulos A, Borrazzo C, Alessi F, Di Nicolantonio C (2021). Post-traumatic Stress Disorder Among COVID-19 Survivors at 3-Month Follow-up After Hospital Discharge. *Journal of General Internal Medicine*.

[5] Boisclair Demarble J, Fortin C, D’Antono B, Guay S (2020). Gender Differences in the Prediction of Acute Stress Disorder from Peritraumatic Dissociation and Distress Among Victims of Violent Crimes. *Journal of Interpersonal Violence*.

[6] Parker C, Shalev D, Hsu I, Shenoy A, Cheung S, Nash S (2021). Depression, Anxiety, and Acute Stress Disorder Among Patients Hospitalized With COVID-19: A Prospective Cohort Study. *Journal of the Academy of Consultation-Liaison Psychiatry*.

[7] Edgelow M, Harrison L, Miceli M, Cramm H (2020). Occupational therapy return to work interventions for persons with trauma and stress-related mental health conditions: A scoping review. *Work (Reading, Mass.)*.

[8] Tamayo-Gómez A, Velásquez-Suárez J, Páramo-Duque L, Ortiz-Carmona D, Escobar-Gómez L, Cortés-López V (2022). Epidemiology and factors associated with Acute Stress Disorder in burned patients: a case-control study. *Burns: Journal of the International Society for Burn Injuries*.

[9] Cox CE, Gu J, Ashana DC, Pratt EH, Haines K, Ma J (2023). Trajectories of Palliative Care Needs in the ICU and Long-Term Psychological Distress Symptoms. *Critical Care Medicine*.

[10] Kang JH, Yang S (2022). A Therapist’s Vicarious Posttraumatic Growth and Transformation of Self. *Journal of Humanistic Psychology*.

[11] Geoffrion S, Goncalves J, Robichaud I, Sader J, Giguère CÉ, Fortin M (2022). Systematic Review and Meta-Analysis on Acute Stress Disorder: Rates Following Different Types of Traumatic Events. *Trauma, Violence & Abuse*.

[12] Connor KM, Davidson JR (2003). Development of a new resilience scale: the Connor-Davidson Resilience Scale (CD-RISC). *Depression and Anxiety*.

[13] Wang J, Chen Y, Wang YB, Liu XH (2011). Post-traumatic growth rating Scale revision and reliability and validity analysis. *Journal of Nursing*.

[14] Mongodi S, Salve G, Tavazzi G, Politi P, Mojoli F, COVID-19 Post-ICU team (2021). High prevalence of Acute Stress Disorder and persisting symptoms in ICU survivors after COVID-19. *Intensive Care Medicine*.

[15] Chapa-Koloffon GDC, Jean-Tron MG, Ávila-Hernández AV, Márquez-González H, Garduño-Espinosa J (2021). Frequency of Acute Stress Disorder in health care workers of a tertiary level pediatric hospital during the National Safe Distance Strategy for COVID-19 prevention. *Boletin Medico Del Hospital Infantil De Mexico*.

[16] Barnes S, Broom M, Jordan Z (2021). Incidence and prevalence of Acute Stress Disorder and post-traumatic stress disorder in parents of children hospitalized in intensive care units: a systematic review protocol. *JBI Evidence Synthesis*.

[17] Cui K, Sui P, Zang X, Sun Y, Liu X (2022). Development and validation of a risk prediction model for post-traumatic stress disorder symptoms in patients with acute myocardial infarction in China. *Annals of Palliative Medicine*.

[18] Smith P, Dalgleish T, Meiser-Stedman R (2019). Practitioner Review: Posttraumatic stress disorder and its treatment in children and adolescents. *Journal of Child Psychology and Psychiatry, and Allied Disciplines*.

[19] Ontario Health (Quality) (2021). Internet-Delivered Cognitive Behavioural Therapy for Post-traumatic Stress Disorder or Acute Stress Disorder: A Health Technology Assessment. *Ontario Health Technology Assessment Series*.

[20] Fernando SM, Scott M, Talarico R, Fan E, McIsaac DI, Sood MM (2022). Association of Extracorporeal Membrane Oxygenation With New Mental Health Diagnoses in Adult Survivors of Critical Illness. *JAMA*.

[21] Pagan BT, Wyant K, Chien J, Coker KL (2019). Prevalence Rates of Acute Stress Disorder Symptomatology and Association to Juvenile Crime Involvement. *The Journal of the American Academy of Psychiatry and the Law*.

[22] Carter JS, Kearns AM, Vollmer KM, Garcia-Keller C, Weber RA, Baker NL (2020). Long-term impact of acute restraint stress on heroin self-administration, reinstatement, and stress reactivity. *Psychopharmacology*.

[23] Chen F, Fan W, Li Y (2022). Influence of Psychological Supervision on Athletes’ Compliance, Mental Elasticity Characteristics and Acute Stress Disorder in Traumatic Fracture Rehabilitation Training. *Iranian Journal of Public Health*.

[24] Hoşgelen EI, Alptekin K (2021). Letter to the Editor: THE IMPACT OF THE COVID-19 PANDEMIC ON SCHIZOPHRENIA PATIENTS. *Turk Psikiyatri Dergisi = Turkish Journal of Psychiatry*.

